# Nanocellulose–Carbonized Lignin Films for Enhanced Tetracycline Removal

**DOI:** 10.3390/polym18151806

**Published:** 2026-07-23

**Authors:** Juan Wang, Ziyu Duan, Zhenzhen Zhang, Jungang Jiang, Ying Chang, Zhishun Wei, Jan-Michael Albina

**Affiliations:** 1 Hubei Provincial Key Laboratory of Green Materials for Light Industry, New Materials and Green Manufacturing Talent Introduction and Innovation Demonstration Base, Hubei University of Technology, Wuhan 430068, China; jwang@hbut.edu.cn (J.W.); 2110412427@hbut.edu.cn (Z.Z.); jungang.jiang@hbut.edu.cn (J.J.); cy0025@126.com (Y.C.); wei.zhishun@hbut.edu.cn (Z.W.); 2 University of Chinese Academy of Sciences, Beijing 100049, China; zy.duan@siat.ac.cn

**Keywords:** carbonized lignin, nanocellulose films, water clarification, tetracycline hydrochloride removal, sustainable materials

## Abstract

The development of sustainable and efficient adsorbent materials for tetracycline hydrochloride (TCH) removal is of great importance for wastewater treatment. Structural characterizations (XRD and FTIR) confirmed successful lignin carbonization and the abundance of oxygen-containing functional groups, while N_2_ physisorption revealed a phosphoric acid-induced hierarchical porous structure rich in mesopores to facilitate mass transfer. CL exhibited excellent tetracycline hydrochloride (TCH) adsorption, achieving a maximum capacity of 21.06 mg g^−1^ within 2 h. The adsorption data closely followed the pseudo-second-order kinetic model, indicating a predominantly chemisorption process. While nanocellulose enabled the fabrication of flexible, self-supporting films with well-dispersed CL, excessive loading deteriorated film integrity. An optimized 30 wt% CL content achieved the ideal balance between structural stability and adsorption performance, demonstrating that these sustainable films hold great potential for aquatic antibiotic remediation.

## 1. Introduction

The widespread use of antibiotics has substantially enhanced the quality of life for humans, animals, and plants owing to their potent antibacterial activity [1]. However, the inefficient biotransformation of antibiotics in humans and animals facilitates their excretion, contributing to their broad occurrence across multiple environmental compartments. Various antibiotic classes, including sulfonamides, quinolones, tetracyclines, macrolides, β-lactams, and chloramphenicols, have been frequently detected in surface water, groundwater, soil, and sediments [2]. Prolonged exposure to antibiotics, even at low concentrations, can exert detrimental effects on the liver [3], kidney [4], immune system [5], and other organs. The persistence of antibiotics such as tetracycline hydrochloride (TCH) in aquatic environments has become a major concern due to their potential to induce antimicrobial resistance and disrupt aquatic ecosystems. TCH, a broad-spectrum antibiotic commonly used in veterinary and human medicine, is frequently detected in surface water and wastewater effluents because of its incomplete metabolism and resistance to biodegradation. Consequently, developing sustainable and efficient adsorbent materials for its removal has attracted considerable attention. Adsorption represents one of the most efficient processes for the removal of contaminants from aquatic systems. Numerous adsorbents have been designed to remove most soluble pollutants, including organic compounds [6], heavy metals [7], etc. Carbonaceous adsorbents have been extensively applied for the adsorption of antibiotics, achieving consistently high removal efficiencies. These adsorbents can be synthesized from diverse agricultural waste materials [8], thereby reducing fabrication costs and contributing to the valorization of solid waste streams. Nanocellulose (NC), derived from renewable biomass, exhibits a high surface area, abundant hydroxyl groups, and tunable surface chemistry, making it a promising material for pollutant adsorption [9,10]. Lignin-based adsorbents have been widely applied to the treatment of wastewater containing heavy metal ions [11], inorganic anions, organic dyes and pharmaceuticals. It was demonstrated that the porous architecture endowed the material with abundant active sites and facilitated ion transport, leading to enhanced adsorption capacity [12]. Meanwhile, lignin-based adsorbents can efficiently capture water pollution through chelation, ion exchange and/or electrostatic interactions; they can be easily modified by versatile physical/chemical methods to fabricate desirable adsorbents with good absorption capacity and selectivity for the target pollutions [13,14]. For example, Yuan et al. reported that a novel fluorescent lignin-based hydrogel, synthesized using cellulose nanofibers and carbon dots, exhibited enhanced adsorption performance toward Cr(VI) [15]. Barker-Rothschild et al. reported that lignin-based porous carbon could be a good adsorbent for CO_2_ capture [16]. Furthermore, lignin modified with metal oxides has shown markedly enhanced adsorption performance. Xiao et al. developed a SiO_2_@sodium lignosulfonate adsorbent with a high specific surface area (293.4 m^2^/g) and porous structure, which increased the exposure of active adsorption sites; however, prolonged contact times were required to achieve effective adsorption [17]. A MnO_2_ nanodot–modified lignin nanocomposite (MnO_2_@LNP), exhibiting high methylene blue adsorption (806 mg/g), was synthesized by Zhai et al. MnO_2_ modification reduced particle size (30–70 to 10–30 nm), increased surface area (51.69 m^2^/g), and enhanced thermal stability and separability compared with LNP [18]. An organosolv-lignin and aluminum pillared bentonite (Al-bent)-supported nZVI (L-nZVI@Ab) was synthesized by Chi et al., exhibiting a Cr(VI) removal capacity of 46.2 mg/g [19]. A novel amino-functionalized lignin-based biosorbent, with maximum adsorption capacities of 74.84, 54.20, 53.12 and 49.42 mg/g for Ni^2+^, Cd^2+^, As(V) and Cr(VI) ions, respectively, was synthesized by Popovic et al. [20]. Owing to its 3D amorphous structure, functional groups such as phenolic hydroxyl and methoxy, and inherent biocompatibility, lignin is regarded as a potential adsorbent material [21]. Lignosulfonate and other modified lignin including chemical modification, nano-particles and lignin hydrogel indicated high potential as heavy metal absorbers [22].

Currently, most research studies are based on chemical reactions such as esterification, etherification, halogenation, and oxidation, which connect active functional groups that can effectively bind heavy metal ions to the cellulose skeleton to improve the adsorption capacity of lignocellulose. Meanwhile, many studies have used lignin as a raw material, making it a fundamental material for porous materials, magnetic spheres, and other applications [23]. Lignin/Fe_3_O_4_ magnetic spheres exhibited high adsorption capacities of 100.00 mg/g, 353.36 mg/g, 223.71 mg/g and 180.18 mg/g for Cu (II), Ni (II), Cr (VI) and methylene blue (MB), respectively, with adsorption equilibrium attained within 60 min [24]. A robust aminated cellulose-based porous adsorbent (PGPW) developed by Zhu from delignified wood and amino-rich polymer exhibited excellent adsorption capacity (188.68 mg/g) for Cu(II) [25]. Previous studies have reported that biomass waste-derived activated carbon is effective in adsorbing commonly encountered metal contaminants, including Cr, Pb, Cu, Cd, Hg, and As [26]. Lignocellulosic films can also be used as potential bioplastics due to their strong, biodegradable properties [27]. Makarov et al. demonstrated that low-alpha-cellulose-based membranes exhibited a dense structure. The membrane prepared from bleached pulp achieved a maximum tensile strength of 5.7 MPa and an elastic modulus of 6.4 GPa. Moreover, residual lignin impurities increased the water contact angle to 48° and improved the membrane’s rejection performance for anionic dyes [28]. CL serves as the functional adsorption component, while nanocellulose acts as a supporting matrix and binder. The incorporation of carbonized lignin introduces additional adsorption sites and improves the adsorption performance while maintaining the integrity of the self-supporting film.

In this work, NCL films with varying mass fractions of nanocellulose and carbonized lignin were synthesized. Their adsorption performance toward tetracycline hydrochloride in aqueous solution was systematically evaluated, and the adsorption mechanism was elucidated using the pseudo-first-order (PFO) and pseudo-second-order (PSO) kinetic models.

## 2. Materials and Methods

### 2.1. Materials

Analytical-grade oxalic acid dihydrate and 1,4-dioxane were obtained from Sinopharm Chemical Reagent Co., Ltd. (Shanghai, China), while analytical-grade phosphoric acid was obtained from Shanghai Aladdin Biochemical Technology Co., Ltd. (Shanghai, China). Cellulose (Cellic^®^CTec3, 160 FPU/mL) was generously provided by Novozymes (Beijing, China).

### 2.2. Synthesis of the Nanocellulose

**Figure 1:**
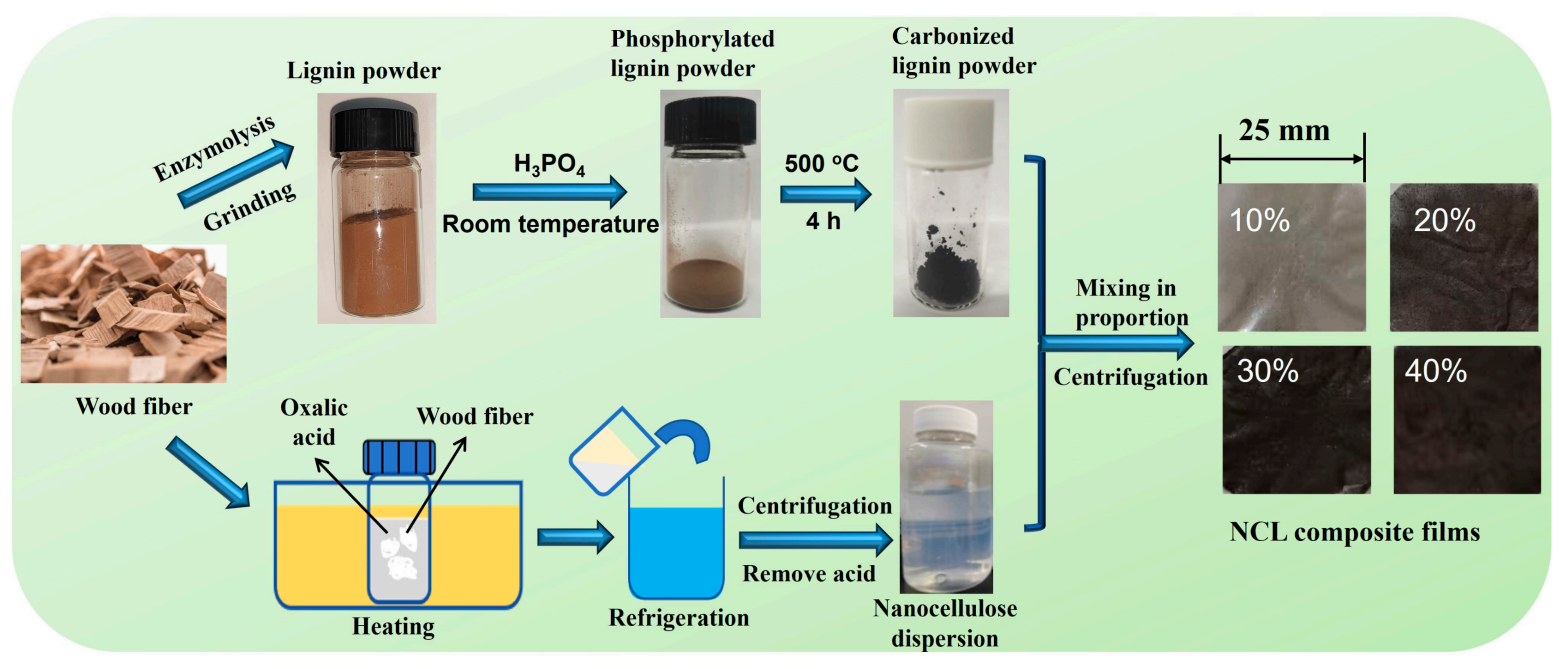
Schematic diagram of the preparation process for the nanocellulose–carbonized-lignin films (NCL).

As shown in Figure 1, oxalic acid crystals (50 g) were heated in a lidded glass bottle at 110 °C under continuous stirring until a transparent liquid formed. After this, 1 g of bleached softwood kraft pulp (BSKP) was added. The sealed mixture was heated and stirred for 30 min, and the resulting hot reaction mixture was subsequently poured into approximately 150 mL of deionized water. After cooling, the suspension was allowed to settle, and the supernatant liquid (yellow) was decanted. This washing process with deionized water was repeated until the supernatant became nearly colorless. A small amount of 1,4-dioxane was then added, and the resulting suspension was subjected to vacuum filtration through a microporous membrane. The solid residue was repeatedly washed with deionized water until the pH of the filtrate reached 6~7. The product was then transferred into a dialysis bag and dialyzed with deionized water for 7 days.

Following dialysis, the suspension was divided equally into two bottles and sonicated alternately using an ultrasonic cell grinder for 30 min, with each bottle treated twice. After centrifugation, the supernatant was collected as the nanocellulose dispersion. If large particles were detected, further ultrasonic treatment was applied. The solid content of the dispersion was determined and, when necessary, increased by mild distillation to reach a target concentration of 0.3–0.7 wt%.

### 2.3. Synthesis of the Lignin, Phosphorylated Lignin (PL) and Carbonized Lignin (CL)

Lignin samples were isolated via a double ball-milling-assisted enzymatic hydrolysis (DEL) method, as described by Chen et al. [29]. The procedure was modified as follows: The benzyl alcohol-extracted raw material was first subjected to ball milling for 60 h. The resulting powder was then mixed with sodium acetate buffer (pH~4.8) at a solid-to-liquid ratio of 1:20 (g/mL), followed by the addition of cellulase (50 FPU/g substrate). The mixture was enzymatically hydrolyzed in a water bath shaker at 50 °C for 48 h. After enzymatic hydrolysis, the mixture was centrifuged, and the residue was thoroughly washed with sodium acetate buffer (pH~4.8) to remove enzymes and hydrolyzed sugars, then freeze-dried. The dried residue was subsequently ball-milled again for 24 h, followed by a second enzymatic hydrolysis using the same conditions. The final residue was washed with acidic water (pH = 2.0), freeze-dried, and ground to obtain lignin powder.

Subsequently, 4 g of lignin powder was mixed with 200 mL of phosphoric acid (66 wt%) and stirred magnetically at room temperature for 4 h. The mixture was subsequently diluted with an appropriate amount of deionized water and centrifuged to collect the precipitate, which was thoroughly washed and dried to obtain phosphorylated lignin (PL, dark-brown powder). Subsequently, 2 g of the PL powder was placed in a crucible and calcined in a muffle furnace at 500 °C for 4 h under ambient air, with a heating rate of 100 °C/min. After calcination, the product was washed with demineralized water, ground, and dried to yield carbonized lignin (CL, black powder).

### 2.4. Synthesis of NCL Films

CL powder was initially added to a beaker, followed by the addition of nanocellulose dispersion to obtain a total mass of 60 mg. The mass fraction of CL was adjusted to 10 wt%, 20 wt%, 30 wt%, and 40 wt%, respectively. Subsequently, an appropriate amount of deionized water and a small quantity of 1,4-dioxane were added to improve the dispersion of the CL. The mixture was sonicated for 10 min using an ultrasonic cell grinder to ensure uniform dispersion of the CL powder within the liquid. The resulting suspension was then transferred into a vacuum filtration apparatus equipped with a microporous filter membrane and filtered until completion. The obtained solid film was carefully peeled from the membrane and vacuum-dried to yield NCL films. The following films with various CL contents were formed: NCL-10 (10 wt% CL), NCL-20 (20 wt% CL), NCL-30 (30 wt% CL), and NCL-40 (40 wt% CL), respectively.

### 2.5. Structure Characterization

The structural properties of the sample were characterized by X-ray diffraction (XRD, PANalytical, Empyrean model, Almelo, Netherlands). XRD measurements were carried out using Cu Kα radiation (wavelength λ = 0.154 nm) at a scanning rate of 5°/min over a 2θ angle range of 5–90°. The morphology and elemental composition were analyzed using a field-emission scanning electron microscope (FE-SEM, Hitachi SU8010, Tokyo, Japan), which provided high-resolution imaging of the surface morphology at an accelerating voltage of 5 kV. Before analysis, the surfaces of the samples were coated with a thin layer of gold (Au). Fourier transform infrared spectroscopy (FTIR) was performed using an INVENIO-S Tensor II system (Bruker Optics Pty. Ltd., Billerica, MA, USA). Freeze-dried PL, CL, and NCL films were analyzed over a spectral range of 400–4000 cm^−1^ using a diamond crystal. Each spectrum was obtained by averaging 64 scans at a resolution of 4 cm^−1^. Elemental compositions (C, H, and N) of lignin, PL, and CL were analyzed using an Elementar UNICUBE elemental analyzer (Elementar Analysensysteme GmbH, Langenselbold, Germany) in CHN mode to monitor changes after phosphoric acid modification and carbonization. Textural properties, including specific surface area and pore structure, were determined via $N_{2}$ adsorption–desorption measurements at 77 K on a Micromeritics ASAP 2460 analyzer (Micromeritics Instrument Corporation, Norcross, GA, USA). Before measurement, samples were degassed in vacuo at 120 °C for 8–10 h. The specific surface area was derived using the Brunauer–Emmett–Teller (BET) method. The total pore volume and pore size distribution were calculated from the nitrogen adsorption at $P/P_{0}\approx 0.99$ and the Barrett–Joyner–Halenda (BJH) method (based on the desorption branch), respectively.

### 2.6. Adsorption and Kinetic Experiments

To evaluate adsorption performance, the adsorption capacities of various samples for tetracycline hydrochloride were measured under the same experimental conditions. The tetracycline hydrochloride solution had an initial concentration of about 30 mg/L, with 100 mL of solution and 100 mg of sample used in each test. Adsorption experiments were performed at room temperature under continuous stirring for 2 h, with the solution concentration monitored every 30 min. The adsorption performance of the composite films was tested using a tetracycline hydrochloride solution (initial concentration ≈ 30 mg/L, solution volume of 50 mL and sample mass of 50 mg). The shredded composite films were added to the tetracycline hydrochloride solution at room temperature and stirred continuously to facilitate adsorption. The adsorption lasted for 2 h, with the solution concentration measured every 30 min to determine the adsorption abilities of the samples.

#### 2.6.1. Linear Forms of the Isotherm Models

The amount of tetracycline hydrochloride adsorbed at equilibrium (*q*_e_, mg/g) was calculated following Equation (1):

(1) $$q_{e}=\frac{\left(C_{0}-C_{e}\right)\times V}{m}$$

where *C*_0_ and *C*_e_ (mg/L) are the initial and equilibrium concentrations of tetracycline hydrochloride, *V* (L) is the volume of the tetracycline hydrochloride solution and *m* (g) denotes the mass of the adsorbent.

#### 2.6.2. Adsorption Kinetics Models

The adsorption kinetics of the target species were analyzed using both pseudo-first-order (PFO) and pseudo-second-order (PSO) models to fit the experimental data. The linearized form of the PFO model was originally proposed by Lagergren in 1898 [30]. It assumes that the adsorption rate is proportional to the number of unoccupied adsorption sites and is generally used to describe physisorption-dominated processes. The PFO equation is

(2) $$\ln(q_{e}-q_{t})=\ln q_{e}-k_{1}t$$

where *q*_e_ and *q*_t_ (mg/g) denote the adsorption capacities of tetracycline hydrochloride at equilibrium and at a given time, respectively. Equation (2) is widely used to fit the kinetics data and to calculate parameters *q*_e_ and *k*_1_ by plotting ln(*q*_e_ − *q*_t_) vs. time.

The PSO model, in contrast, assumes that the adsorption rate is proportional to the square of the number of available active sites and is often used to describe chemisorption-controlled processes. The PSO differential equation is

(3) $$\frac{t}{q_{t}}=\frac{1}{k_{2}{q_{e}}^{2}}+\frac{t}{q_{e}}$$

where *k*_2_ (g·mg^−1^·min^−1^) is the PSO rate constant.

## 3. Results and Discussion

### 3.1. Structural Characterization

**Figure 2:**
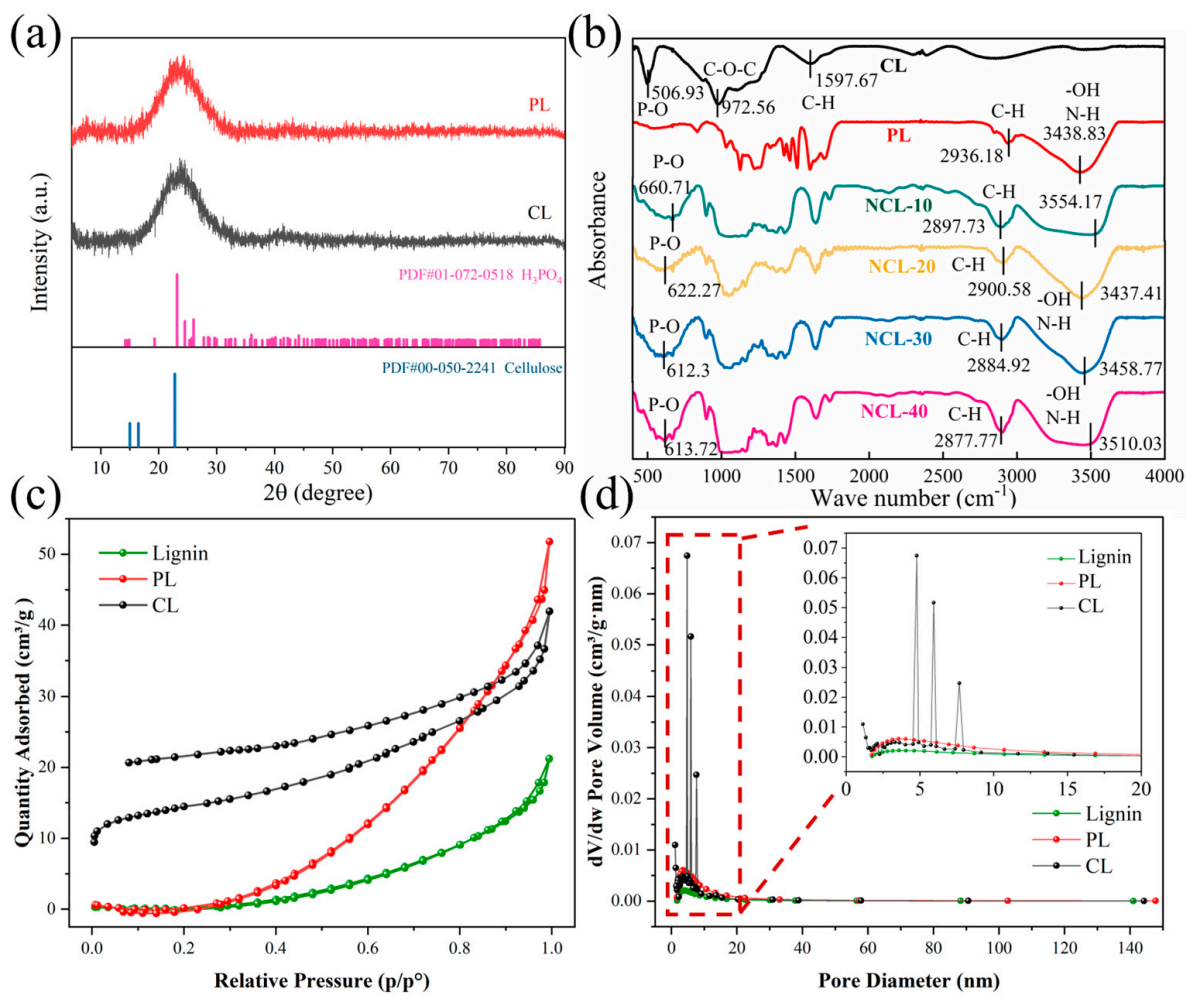
(**a**) XRD analysis of phosphorylated lignin (PL) and carbonized lignin (CL); (**b**) FTIR spectrum for CL, PL, NCL films; (**c**) N2 adsorption–desorption isotherms; and (**d**) the corresponding pore size distribution curves of the synthesized lignin, PL and CL samples.

Figure 2a presents X-ray diffraction (XRD) patterns of PL and CL powder. Both samples exhibit only a broad diffraction halo in the 20–30° region, centered at approximately 23°, with no distinct crystalline peaks. This characteristic indicates that PL and CL are predominantly amorphous, possessing short-range structural order but lacking long-range periodicity. The broad reflection around 23° is likely associated with residual cellulose-derived structures and phosphorus-containing species formed during phosphoric acid activation. The absence of sharp diffraction peaks suggests that the phosphoric acid treatment and subsequent high-temperature carbonization disrupted the original ordered structure of lignin, thereby inhibiting the formation of crystalline domains. Concurrently, phosphorus incorporation and oxidation introduced phosphate/phosphoric ester groups (-PO_4_ and P-O-C) and oxygen-containing functionalities (e.g., C=O), as confirmed by FTIR and XPS analyses. These chemical and structural modifications collectively intensified the disordered nature of PL and CL, yielding highly amorphous frameworks.

Figure 2b presents the FTIR analysis of CL, PL, and NCL composite films (NCL-10 to NCL-40), which reveals multiple characteristic absorption bands, reflecting the retention of aromatic frameworks and oxygen-containing functional groups after processing. For CL, a prominent band at 1597.67 cm^−1^ is assigned to aromatic C=C skeletal stretching vibrations, indicating the preservation of lignin-derived aromatic structures after carbonization. The peak at 972.56 cm^−1^ corresponds to out-of-plane C–H bending of the aromatic ring or C–O–C vibrations, reflecting residual aromatic ether linkages or substituted structures. The absorption at 506.93 cm^−1^ belongs to the low-wavenumber region and may arise from skeletal vibrations or bending modes associated with surface oxygen-containing functional groups. For PL, the broad band at 3438.83 cm^−1^ is attributed to hydrogen-bonded O–H stretching vibrations from phenolic and alcoholic hydroxyls as well as adsorbed moisture. The peak at 2936.18 cm^−1^ corresponds to aliphatic C–H stretching vibrations (CH_2_/CH_3_), while the 970–1600 cm^−1^ region includes out-of-plane C–H bending and aromatic C=C skeletal vibrations, indicating the retention of aromatic skeletons and residual organic moieties. In the NCL films, the broad band at 3450–3560 cm^−1^ is attributed to hydrogen-bonded O–H stretching, whereas the peaks at 2930–2870 cm^−1^ are assigned to aliphatic C–H stretching vibrations. The absorption observed at 610–630 cm^−1^ may arise from out-of-plane C–H bending of the aromatic ring or bending vibrations related to skeletal and surface oxygen-containing groups. Overall, these observations indicate that while the samples are mainly amorphous carbon, residual aromatic structures and oxygen-containing functional groups remain, providing active sites for adsorption.

The nitrogen adsorption–desorption isotherms and pore size distributions of lignin, PL, and CL are presented in Figure 2c,d. Pristine lignin exhibits a relatively low nitrogen uptake throughout the entire relative pressure range, reflecting its limited specific surface area and pore volume. After phosphoric acid phosphorylation, the adsorption capacity of PL increases markedly, especially at high relative pressures, indicating that chemical activation promotes pore development. Following carbonization, CL shows the highest nitrogen adsorption capacity, accompanied by a pronounced hysteresis loop, characteristic of a mesoporous carbon material. The BET analysis quantitatively confirms the evolution of the porous structure. As summarized in Table 1, the specific surface area increases from 2.52 m^2^ g^−1^ for lignin to 3.38 m^2^ g^−1^ for PL and further increases dramatically to 52.07 m^2^ g^−1^ after carbonization, representing an approximately 20-fold increase compared with pristine lignin. Meanwhile, the total pore volume increases from 0.0324 cm^3^ g^−1^ for lignin to 0.0790 cm^3^ g^−1^ for PL due to phosphoric acid activation and remains relatively high (0.0638 cm^3^ g^−1^) after carbonization. Notably, the average pore diameter decreases substantially from 51.44 nm (lignin) and 93.43 nm (PL) to 4.90 nm for CL, indicating that carbonization converts the loosely distributed macroporous structure into a well-developed mesoporous network.

As shown in Figure 2d, lignin exhibits only a limited pore volume with a broad pore size distribution. Phosphoric acid activation increases the pore volume and generates larger pores, whereas subsequent carbonization transforms these large pores into abundant mesopores predominantly distributed between 3 and 10 nm. The substantial increase in specific surface area together with the formation of a uniform mesoporous network is expected to expose more accessible adsorption sites and facilitate the diffusion of tetracycline molecules within the porous structure. Consequently, the synergistic effects of phosphoric acid activation and carbonization significantly improve the textural properties of lignin, providing a favorable porous framework for the enhanced adsorption performance of the NCL composite films.

**Figure 3:**
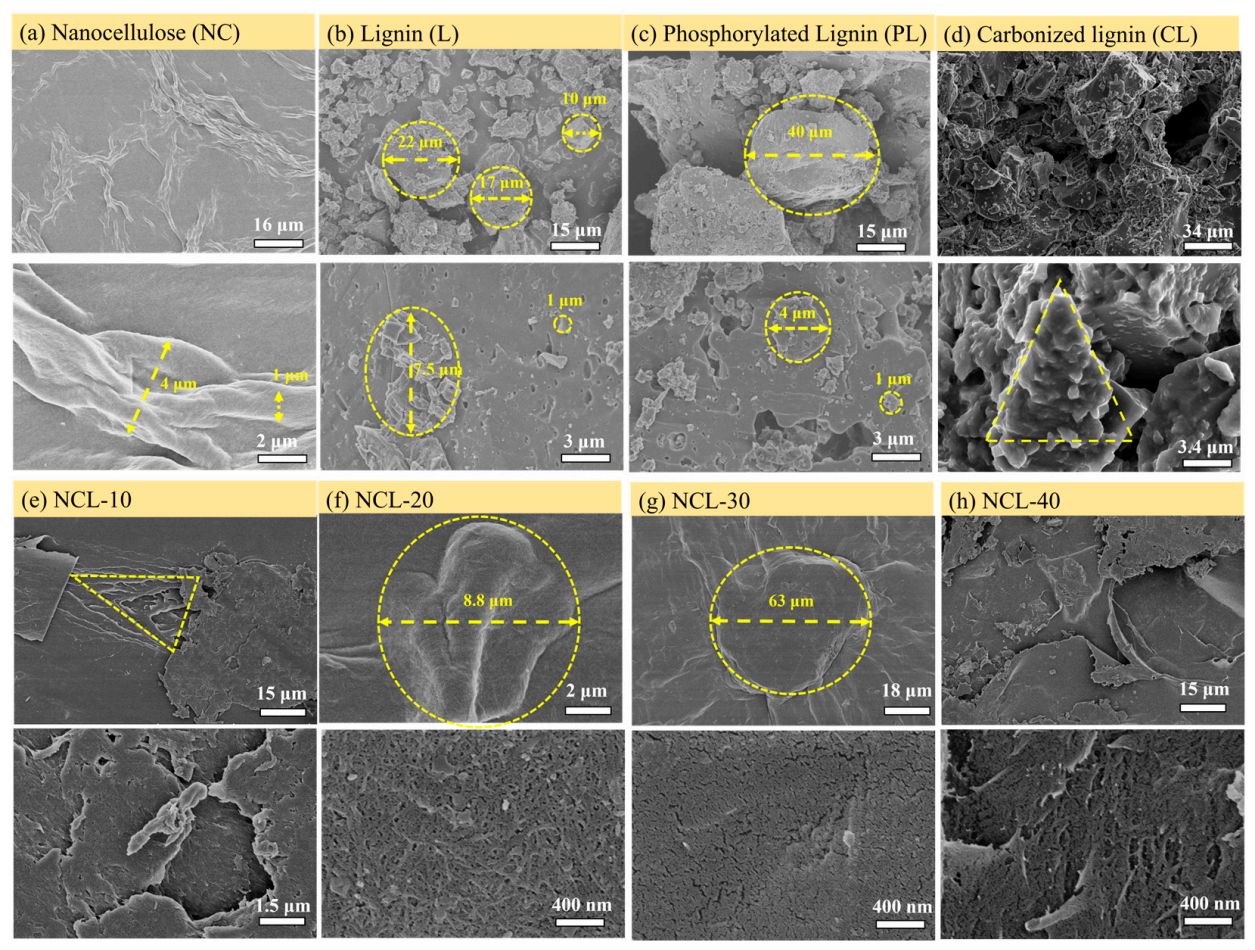
FE-SEM micrographs of the samples: (**a**) NC suspension; (**b**) lignin; (**c**) PL; (**d**) CL powder. FE-SEM micrographs of the composite films: (**e**) NCL-10; (**f**) NCL-20; (**g**) NCL-30; (**h**) NCL-40.

Figure 3a–d presents the surface morphologies of the samples characterized by field emission scanning electron microscopy (FESEM): (a) nanocellulose (NC) suspension, (b) lignin, (c) PL, and (d) CL powder. The FESEM micrographs of the composite films are shown in Figure 3e–h, corresponding to NCL-10 (10 wt% CL), NCL-20 (20 wt% CL), NCL-30 (30 wt% CL), and NCL-40 (40 wt% CL), respectively. As shown in Figure 3a, the nanocellulose exhibits nanocrystal-like structures with lengths ranging from 1 to 4 μm. In Figure 3b, some dark irregularly shaped particles are observed, which are presumed to be lignin aggregates with diameters ranging from approximately 1 to 22 μm. Figure 3c shows that PL consists of dark, irregularly shaped particles with diameters ranging from 1 to 40 μm. After calcination in a muffle furnace at 500 °C for 4 h under an air atmosphere with a heating rate of 100 °C/min, CL was obtained. The morphology of CL (Figure 3d) transformed into a triangular, layered structure. As shown in Figure 3e–h, the surface morphology of the NCL composite films changes noticeably with increasing CL content. The NCL-10 film (Figure 3e) exhibits a relatively smooth and uniform surface, indicating good dispersion of CL within the nanocellulose matrix, although some parts appear to have a triangular, layered structure, which may come from CL. As the CL content increases to 20 wt% (Figure 3f) and 30 wt% (Figure 3g), the surface becomes progressively rougher, and small agglomerates with diameters around 8.8 μm and 63 μm appear, suggesting partial aggregation of CL particles. With increasing CL content, the particle size within the NCL films tends to increase accordingly. At the highest loading (NCL-40, Figure 3h), the surface exhibits pronounced roughness, visible clusters and cracks, implying that excessive CL may lead to reduced interfacial compatibility and non-uniform dispersion within the composite structure.

**Figure 4:**
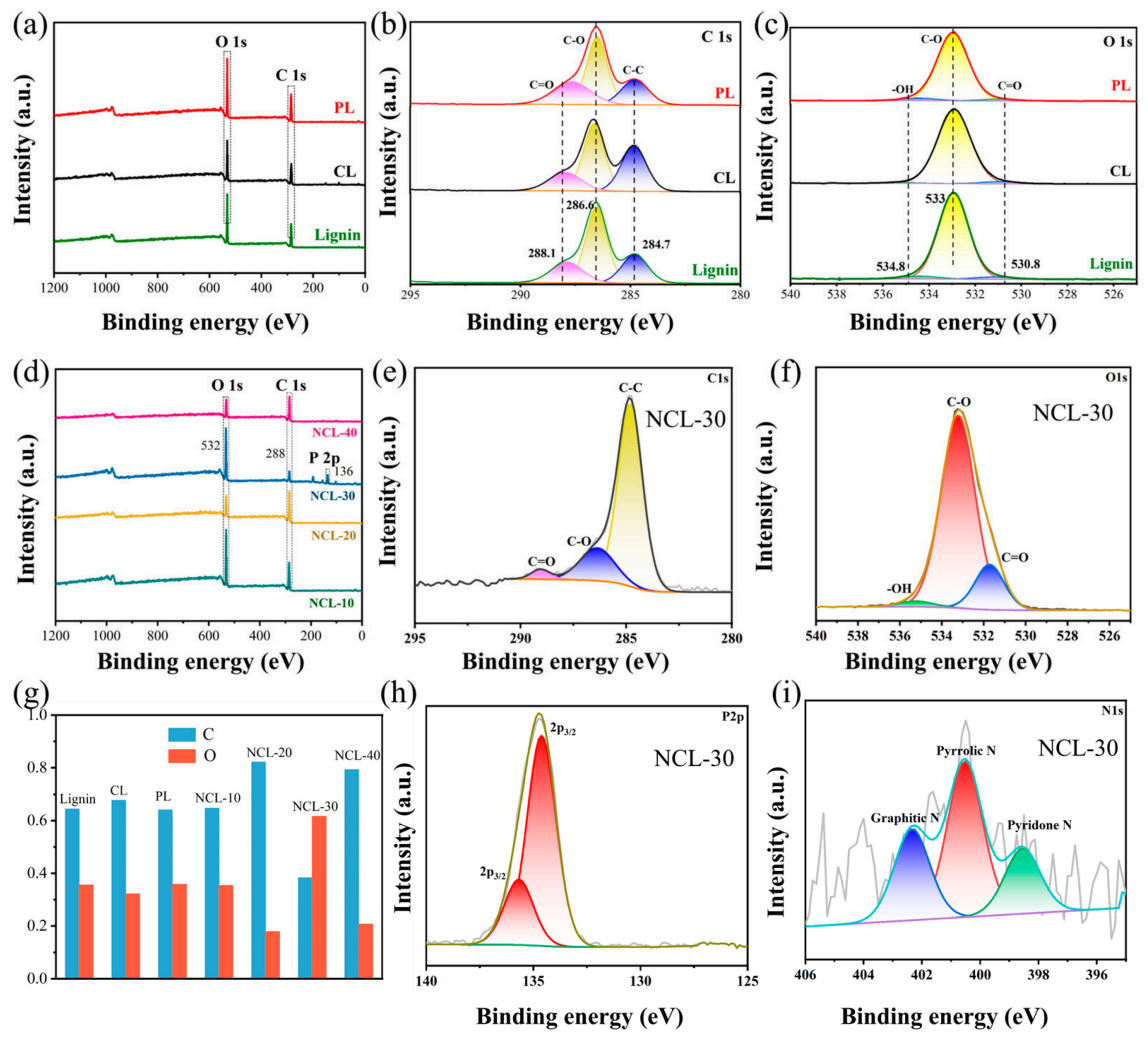
(**a**) Full high-resolution X-ray photoelectron spectrum (XPS) of lignin, CL and PL powder; (**b**) XPS C 1s for lignin, CL and PL powder; (**c**) XPS O 1s for lignin, CL and PL powder; (**d**) full XPS of NCL films (NCL-10, NCL-20, NCL-30, NCL-40); (**e**) XPS C 1s for NCL-30; (**f**) XPS O 1s for NCL-30; (**g**) C and O concentrations for lignin, CL, PL and NCL films; (**h**) XPS P 2p for NCL-30; (**i**) XPS N 1s for NCL-30.

Figure 4 presents the high-resolution X-ray photoelectron spectroscopy (XPS) for all the samples. Figure 4a illustrates the XPS of lignin, CL, and PL powders, in which the dominant core-level peaks corresponding to carbon (C 1*s*, ~285 eV) and oxygen (O 1*s*, ~533 eV) are clearly observed. Figure 4b,c represent the high-resolution C 1*s* and O 1*s* for the three samples. In the C 1*s* spectra, the deconvoluted peaks located at 286.6 eV, 288.1 eV, and 284.7 eV are assigned to C-O, C=O, and C-C/C=C bonds, respectively. For the O 1*s* spectra, the components centered at 533.0 eV, 534.8 eV, and 530.8 eV correspond to C-O, -OH, and C=O species, respectively. Figure 4d presents the XPS spectra of the NCL films, in which the characteristic peaks of C 1*s* (~288 eV) and O 1*s* (~532 eV) are clearly observed. In addition, a P 2*p* signal at approximately 136 eV is detected for the NCL-30 sample. Figure 4e,f,h,i show the high-resolution spectra of the C 1*s*, O 1*s*, P 2*p*, and N 1*s* orbitals of NCL-30. The N 1s signal appears weak and randomly distributed, indicating that nitrogen is essentially absent in the NCL-30 film.

The elemental compositions of lignin, phosphorylated lignin (PL), and carbonized lignin (CL) are presented in Table 2. The carbon content decreased from 59.32 ± 0.10 wt.% for lignin to 55.93 ± 0.20 wt.% after phosphorylation, whereas the hydrogen and nitrogen contents changed only slightly. After carbonization, the carbon content increased significantly to 67.57 ± 0.01 wt.%, while the hydrogen content decreased from 5.18 ± 0.01 wt.% to 2.31 ± 0.01 wt.%. These results indicate that carbonization effectively removed volatile components and promoted the formation of a carbon-rich structure. The nitrogen content also increased to 1.23 ± 0.01 wt.%, reflecting the relative enrichment of nitrogen during pyrolysis. The enhanced carbon content is consistent with the formation of a more condensed aromatic carbon framework, which is favorable for improving the adsorption performance of the carbonized lignin.

### 3.2. Adsorption Performance

**Figure 5:**
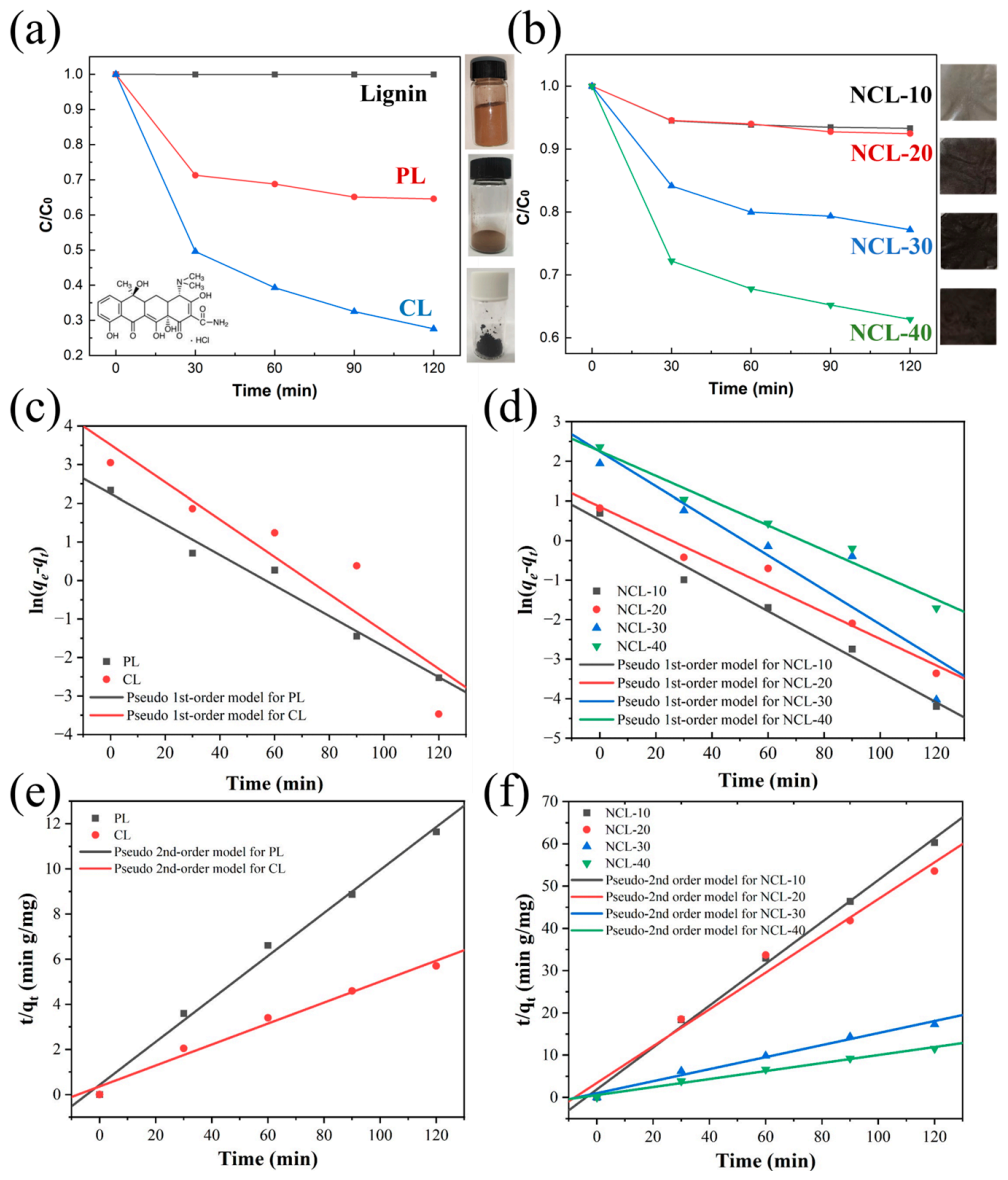
(**a**) *C*/*C*_0_ as a function of time for lignin, PL, and CL powder; (**b**) *C*/*C*_0_ as a function of time for NCL films; (**c**) pseudo-first-order (PFO) kinetic fit for PL and CL; (**d**) PFO kinetic fit for NCL films; (**e**) pseudo-second-order (PSO) kinetic fit for PL and CL; (**f**) PSO kinetic fit for NCL films. The dots are the experimental data.

As illustrated in Figure 5a, the variation in *C*/*C*_0_ with time reflects the adsorption behavior of tetracycline hydrochloride on lignin, PL, and CL powder. The parameter *C*/*C*_0_, defined as the ratio of the solute concentration at a given time (*C*) to its initial concentration (*C*_0_), provides a measure of the relative decrease in tetracycline concentration during the adsorption process. It can be observed that the unmodified lignin exhibited negligible adsorption capacity toward tetracycline hydrochloride. However, following phosphoric acid activation and hydrolysis, the PL displayed a markedly enhanced adsorption performance. Furthermore, upon calcination to obtain CL, the adsorption capacity increased substantially, with the majority of tetracycline hydrochloride molecules being effectively adsorbed within 2 h, corresponding to an adsorption rate of 72.39%. As shown in Figure 5b, the adsorption of tetracycline hydrochloride on NCL films increases with higher CL content, suggesting that CL incorporation enhances active sites and overall affinity toward the antibiotic. Although CL powder exhibits higher adsorption capability than NCL films, powdered adsorbents are difficult to separate from aqueous solutions after adsorption and may cause secondary contamination. Incorporating CL into nanocellulose films provides a self-supporting structure that facilitates handling, recovery, and potential regeneration, thus improving practical applicability. Figure 5c presents ln(*q*_e_ − *q*_t_) as a function of time, describing the adsorption kinetics of tetracycline hydrochloride on PL and CL using the pseudo-first-order (PFO) kinetic model. Compared with PL, CL shows the steepest slope, reflecting a substantially enhanced adsorption rate. Figure 5d illustrates the PFO kinetic model describing the adsorption behavior of tetracycline hydrochloride on NCL composite films. When the mass fraction of CL is relatively low (below 20 wt%), the slopes of the kinetic curves exhibit minimal variation, indicating that the increase in CL content exerts only a minor influence on the adsorption rate. However, a distinct increase in slope is observed when the CL content reaches 30 wt%, suggesting that higher CL loading markedly enhances the adsorption kinetics. These results imply that a small amount of CL contributes little to adsorption enhancement, whereas a higher CL proportion significantly improves the overall adsorption rate of the composite films. Figure 5e,f depict the plots of *t*/*q*_t_ as a function of time, fitted using the pseudo-second-order (PSO) kinetic model for PL, CL, and the NCL composite films. The PSO kinetic model was employed to further evaluate the adsorption kinetics of tetracycline hydrochloride on these materials. It is evident that the PSO kinetic model provides a better fit to the experimental data compared to the PFO model, indicating that the adsorption process is primarily governed by chemisorption involving valence forces through the sharing or exchange of electrons between the adsorbent and tetracycline hydrochloride molecules.

To evaluate the contribution of CL to the overall adsorption performance of the NCL films, the equivalent adsorption efficiency was calculated as follows:

Equivalent adsorption efficiency:

$$\eta =\frac{C_{NCL-x}}{C_{CL}*x\%}$$

where *C*_NCL-x_ is the adsorption capacity for NCL composite films, x is the mass fraction of CL, and *C_CL_* is the adsorption capacity for CL; in this case, *C*_CL_ = 21.06 mg/g.

In the case of the NCL-10 composite film, the following result was obtained:

$$\eta_{NCL-10}=\frac{C_{NCL-10}}{C_{CL}*10\%}=\frac{1.99(mg/g)}{21.06\left(mg/g\right)*10\%}=94.5\%$$

Table 3 presents the adsorption efficiency, adsorption capacity, and equivalent adsorption efficiency of the samples. It is evident that CL exhibits a strong adsorption performance, with an adsorption capacity of 21.06 mg/g and an adsorption efficiency of 72.39%, indicating its superior ability to remove the target compound from aqueous solution. PL shows moderate adsorption performance, with an adsorption capacity of 10.31 mg/g and an adsorption efficiency of 35.43%, suggesting that phosphorylation enhances the adsorption activity of lignin to some extent. In contrast, raw lignin without any modification shows negligible adsorption.

For the NCL series, the adsorption performance varies with the degree of modification. NCL-10 and NCL-20 exhibit relatively low adsorption capacities of 1.99 mg/g and 2.24 mg/g, corresponding to adsorption efficiencies of 6.7% and 7.54%, but their equivalent adsorption efficiencies are relatively high (94.5% and 53.2%), indicating that even a small amount of active CL contributes significantly to the adsorption process. As the CL content increases, the adsorption performance improves: NCL-30 achieves an adsorption capacity of 6.94 mg/g (22.82% efficiency) with an equivalent adsorption efficiency of 109.8%, and NCL-40 reaches 10.41 mg/g (37.07% efficiency) with an equivalent adsorption efficiency of 123.6%. The equivalent adsorption efficiency of NCL-40 exceeding 100% enhances utilization efficiency between cellulose and CL, where the interaction between the two components enhances the overall adsorption capacity beyond the contribution of CL alone. Overall, these results demonstrate that CL is the primary active component for adsorption, and its incorporation into NCL composites significantly enhances adsorption performance. The enhanced utilization efficiency observed in higher-CL-content composites provides valuable guidance for designing efficient lignin-based adsorbents with improved activity.

Table 4 presents a comprehensive comparison of the adsorption capacity of various biomass-derived materials for TCH. As observed, the adsorption capacity of the phosphorylated and carbonized lignin carbon obtained in this work (21.06 mg/g) is lower than some previously reported materials, such as N-doped lignin carbon (1105.9 mg/g) and KOH-activated Chinese medicine residues (786 mg/g). This discrepancy could be mainly attributed to the difference in specific surface area and pore structures. While high-temperature activation with strong alkalis or chemical dopants creates vast micro/mesopores for physical trapping, our phosphorylation process primarily relies on surface-active phosphorus functional groups for targeted chemical interaction, resulting in moderate physical adsorption capacity. Nevertheless, the proposed adsorbent in this work exhibits distinct advantages from a practical and environmental perspective: First, the fabrication process involving phosphorylation and carbonization is highly straightforward, green, and cost-effective, bypassing the need for hazardous activation reagents (e.g., KOH or transition metals) that cause secondary pollution. Second, using lignin—an abundant, low-cost industrial byproduct—as the precursor significantly reduces the overall preparation cost, enhancing its feasibility for large-scale industrial wastewater remediation. Therefore, despite a moderate maximum adsorption capacity, the synthesized phosphorylated lignin carbon represents a more balanced, economically viable, and environmentally friendly candidate for practical applications.

## 4. Conclusions

In summary, CL was successfully prepared through phosphoric acid activation and carbonization. XRD analysis showed that both PL and CL exhibited predominantly amorphous structures, while FTIR confirmed the successful phosphorylation/carbonization process and the presence of oxygen-containing functional groups. Nitrogen adsorption–desorption and pore size distribution analyses demonstrated that phosphoric acid activation effectively improved the pore structure of lignin, generating abundant mesopores with a small proportion of micropores, which provided favorable adsorption sites and mass transfer pathways. Hence, CL exhibited an adsorption capacity of 21.06 mg g^−1^ toward tetracycline hydrochloride within 2 h. Nanocellulose enabled the uniform dispersion of CL and the fabrication of flexible self-supporting films by simple vacuum filtration. However, excessive CL loading reduced film integrity and homogeneity, while a CL content of 30 wt% provided the best balance between structural stability and adsorption performance. The pseudo-second-order (PSO) model described the adsorption kinetics better than the pseudo-first-order (PFO) model, suggesting that the adsorption process may involve chemical interactions. These findings demonstrate that NCL films are promising sustainable adsorbents for antibiotic removal from wastewater.

## Figures and Tables

**Table 1:** BET surface area and pore structure parameters of lignin, PL, and CL.

Sample	BET Surface Area (m^2^/g)	Total Pore Volume (cm^3^/g)	Average Pore Diameter (nm)
Lignin	2.5164	0.032363	51.4428
PL	3.3816	0.078986	93.4301
CL	52.0654	0.063787	4.9

**Table 2:** Elemental compositions of lignin, phosphorylated lignin (PL), and carbonized lignin (CL).

Name	C (wt.%)	H (wt.%)	N (wt.%)
Lignin	59.32 ± 0.10	5.18 ± 0.04	0.77 ± 0.01
PL	55.93 ± 0.20	5.178 ± 0.01	0.7 ± 0.02
CL	67.57 ± 0.01	2.31 ± 0.01	1.23 ± 0.01

**Table 3:** Adsorption efficiency, adsorption capacity and equivalent adsorption efficiency for the samples.

Samples	Adsorption Efficiency (%)	Adsorption Capacity (mg/g)	Equivalent Adsorption Efficiency (%)
Lignin	0	0	-
PL	35.43	10.31	-
CL	72.39	21.06	-
NCL-10	6.7	1.99	94.5
NCL-20	7.54	2.24	53.2
NCL-30	22.82	6.94	109.8
NCL-40	37.07	10.41	123.6

**Table 4:** Performance comparison of different biomass-derived adsorbents for adsorption capacity.

Biomass	Method	Adsorption Capacity (mg/g)	Ref. (Year)
**L** **ignin carbon**	**P** **hosphorylated and carbonized**	**21.06**	**This work**
Lignin carbon	N-doped	1105.9	Li et al. (2025) [31]
Lignin	Magnetized with NiFe_2_O_4_	714.28	Kargar Samani et al. (2025) [32]
Crayfish shell	Ball-milled biochar	39.1	Zhang et al. (2021) [33]
Lignin carbon	Hydrothermal–phosphoric acid synergistic activation	219.81	Li et al. (2026) [34]
Corncob biochar	Phosphoric acid-activated	953.62	Zhao et al. (2026) [35]
Chinese medicine residues	Hydrothermal and KOH treatment	786	Xu et al. (2026) [36]
Corn stalk and wheat straw	Lignin impregnation	31.48	Xiang et al. (2022) [37]

## Data Availability

The original contributions presented in this study are included in the article. Further inquiries can be directed to the corresponding author.

## Abbreviations

The following abbreviations are used in this manuscript:

NCF	Nanocellulose–carbonized lignin films
CL	Carbonized lignin
TCH	Tetracycline hydrochloride
FE-SEM	Field emission scanning electron microscopy
PFO	Pseudo-first-order
PSO	Pseudo-second-order
